# Embodying pervasive discrimination: a decomposition of sexual orientation inequalities in health in a population-based cross-sectional study in Northern Sweden

**DOI:** 10.1186/s12939-017-0522-1

**Published:** 2017-01-21

**Authors:** Per E Gustafsson, Ida Linander, Paola A Mosquera

**Affiliations:** Department of Public Health and Clinical Medicine, Unit of Epidemiology and Global Health, Umeå University, SE-901 87 Umeå, Sweden

**Keywords:** Sexual orientation, LGBQ, Health inequality, Mental health, Self-reported health, Decomposition analysis

## Abstract

**Background:**

Studies from Sweden and abroad have established health inequalities between heterosexual and non-heterosexual people. Few studies have examined the underpinnings of such sexual orientation inequalities in health. To expand this literature, the present study aimed to employ decomposition analysis to explain health inequalities between people with heterosexual and non-heterosexual orientation in Sweden, a country with an international reputation for heeding the human rights of non-heterosexual people.

**Methods:**

Participants (*N* = 23,446) came from a population-based cross-sectional survey in the four northernmost counties in Sweden in 2014. Participants completed self-administered questionnaires, covering sexual orientation, mental and general physical health, social conditions and unmet health care needs, and sociodemographic data was retrieved from total population registers. Sexual orientation inequalities in health were decomposed by Blinder-Oaxaca decomposition analysis.

**Results:**

Results showed noticeable mental and general health inequalities between heterosexual and non-heterosexual orientation groups. Health inequalities were partly explained (total explained fraction 64-74%) by inequalities in degrading treatment (24-26% of the explained fraction), but to a considerable degree also by material conditions (38-45%) and unmet care needs (25-43%).

**Conclusions:**

Psychosocial experiences may be insufficient to explain and understand health inequalities by sexual orientation in a reputedly ‘gay-friendly’ setting. Less overt forms of structural discrimination may need to be considered to capture the pervasive material discrimination that seems to underpin the embodiment of sexual minority inequalities. This ought to be taken into consideration in research, policy-making and monitoring aiming to work towards equity in health across sexual orientations.

## Background

An increasing number of reports in Europe and abroad have demonstrated disparities in various forms of health between people with heterosexual and non-heterosexual orientation [1, 2], and the need for increased research on this topic has recently been called for [3]. The present study seeks to contribute to this task by examining the underpinnings of sexual orientation-based inequalities in self-assessed health in Sweden, a setting which has an international reputation of heeding the human rights of people with non-heterosexual orientation.

Sexual orientation-based inequalities in health have commonly been framed within *minority stress theory* [4], which focuses on differential exposure to stressors, such as experience of sexuality-based discrimination [5], in minority and majority sexual orientation groups, and on the process whereby this in turn affects health. Here, majority sexual orientation usually refers to heterosexual orientation, with minority orientation commonly encompassing lesbian/gay/bisexual, and sometimes also queer or questioning orientations (collectively referred to as LGBQ in the present report). Moreover, *fundamental cause theory* [6], which emphasizes the unequal distribution of health-protective resources such as knowledge, prestige, and power as underpinning health inequalities, has also been used to explain sexual orientation inequalities in health [7]. From the point of departure of the more general *ecosocial theory* [8], minority stress and fundamental cause theories could jointly be framed as an expression of embodiment [9, 10]; the process whereby structural social inequalities (e.g., unequal distribution of power and resources based on sexual orientation) through multiple pathways (e.g., discriminatory experiences) become differentially embodied in health in LGBQ and heterosexual people, thereby creating and upholding sexual orientation health inequalities in the population.

Sweden has internationally been recognized for offering a favorable human rights situation for LGBQ people, e.g., with respect to anti-discrimination laws, parental rights, legislation on homophobic hate crimes, and the population’s attitudes towards LGBQ people [11–13]. However, in recent years, Sweden has not improved in this respect to the same degree as other European countries, and has moved from being number one on The International Lesbian, Gay, Bisexual, Trans and Intersex Association’s (ILGA) European country ranking in 2010, to 12th place in 2016 [11, 14]. The ILGA report from 2016 for example notes that a new Swedish law will hinder LGBQ asylum seekers, and that several religious leaders and right-wing politicians have made homophobic public statements [14]. As such, Sweden in the mid-2010s may represent an internationally interesting setting undergoing a transition from a leading to a moderate position in terms of ‘gay-friendliness’. This development would be expected to impact on sexual orientation inequalities.

Recent reports from Sweden have showed that LGBQ persons indeed experience more discrimination and victimization than do heterosexual persons, and that they also display worse self-reported health and preventable morbidity [2, 7]. Investigations attempting to explain sexual orientation inequalities in health have suggested that individual-level discrimination plays a role, such as experiences of humiliating treatment, threats and violence [15], which is in line with the differential exposure to psychosocial stressors described by minority stress theory. However, little attention has been paid to less overt structural-level discrimination; here referring to societal conditions constraining the individual’s possibilities, resources and well-being [16]. Such barriers can for example involve getting turned down from a job, receiving a smaller raise than one’s colleagues, or challenges to accessing or receiving health care [17]; neither of which may be readily distinguishable as discrimination by the individual, but which nevertheless can exert important health effects in the long-run. This depiction of sexual orientation inequalities in health, underpinned by a multitude of subtle and flexible resources that can be leveraged by those of the sexual orientation majority but are lacking for those of sexual minority, instead corresponds more closely to fundamental cause theory.

Another limitation of previous research is the dominance of conventional regression models [2, 15], which are illustrative for predicting health but give little in the way of uncovering what underpins *inequalities* in health. Decomposition techniques [18, 19], still fairly uncommon within public health, offer here an alternative approach which not only predicts the unidimensional construct of health, but instead attributes sources of the compound construct of social inequality in health to independent conditions.

The present population-based study from Northern Sweden aims to decompose the mental and general physical health inequalities between heterosexual and LGBQ sexual orientation groups, by conditions and experiences signifying both individual-level psychosocial factors which are reflecting minority stress theory, as well indicators or more subtle structural-level discrimination that are indicative of fundamental cause theory.

## Methods

### Study population and procedures

The sample came from the cross-sectional Health on Equal Terms (HET) survey conducted in 2014 by the four northernmost counties of Sweden; Norrbotten, Västerbotten, Jämtland-Härjedalen and Västernorrland. The survey represents the regionally expanded sample of the national HET survey, which is implemented in collaboration between the Swedish National Public Health Agency and the individual county councils, with the purpose of monitoring health and living conditions of the population. The target population comprised all residents in the four counties aged between 16 and 84 years of age. A two-stage probabilistic sampling procedure representative of the counties and municipalities was employed, with 50% responding to the survey, resulting in a gross sample of *N* = 25,667. The survey was implemented through postal questionnaires covering areas such as health, health behaviors, health care use and psychosocial and material conditions. In addition, register data on sociodemographic information from 2012, originating in the total population registers of Statistics Sweden, were linked to the survey participants. Due to item non-response on either health outcome or sexual orientation items of the questionnaire, the total sample of the present investigation comprised a maximum of *N* = 844 individuals identifying themselves as LGBQ and *N* = 23,446 as heterosexual (see below), and with effective N varying between analyses, lowest *N* = 22,460 (*N* = 724 LGBQ and *N* = 21,736 heterosexual).

### Measures

#### Outcomes

*Mental health* was measured by the GHQ-12 [20, 21], covering twelve items with four Likert coded response options, with items summed up to form an index (range = 36; Cronbach α = 0.89). *General physical health complaints* were measured by ten general physical symptoms (musculoskeletal pain in neck; back; and extremities; headache; worries; tiredness; sleeping difficulties; eczema; tinnitus; bowel symptoms), which were scored on three-level Likert scales and summed up into an index (range = 20; Cronbach α = 0.72). Mental health and general health correlated of moderate strength with each other (Pearson’s *r* = 0.50).

#### Sexual orientation

Sexual orientation was based on the item ‘What is your sexual orientation’ with the response options being ‘heterosexual’, labeled *Heterosexual orientation* (*N* = 23,466), ‘bisexual’ (*N* = 326), ‘homosexual‘(*N* = 121) and ‘unsure about my sexual orientation’ (*N* = 397). The three non-heterosexual groups were collapsed into one category, labelled *Lesbian-Gay-Bisexual-Questioning (LGBQ) orientation* (*N* = 844), thus forming a binary variable of sexual orientation.

#### Explanatory factors

In addition to demographic factors, explanatory factors were selected with the goal of capturing indicators of discrimination, either concrete, individual-level experiences, e.g., exposure to violence; or conditions which could signify less overt, structural-level discrimination, e.g., problems in getting a job. By this focus, we also sought to avoid factors likely to represent proximal mediators or consequences of the chosen health outcomes, such as health behaviors or feelings of trust which may be entangled with depressive symptoms.

As demographic factors, the following three factors were included:

- *Gender*, coded as man (0) and woman (1).
- *Age*, coded as young adulthood (16–35 years) (0), middle-age (36–65 years) (1), and old age (66–85 years) (2).
- *Country of birth*, coded as Sweden (0) and outside Sweden (1).

Socioeconomic factors covered the following four factors:

- *Income*, measured as annual disposable income and divided into quintiles
- *Education*, coded as low (0), medium (1) and high (2) education.
- *Occupational class*, based on the socioeconomic classification of Statistics Sweden [22] and coded as close as possible to Erikson-Goldthorpe-Portocarero (EGP) scheme [23] into Service class (1), Routine non-manual class (2), Petty Bourgeoisie (3), Assistant non-manual (4), Skilled manual (5), Unskilled manual (6) class, and with those with unspecified occupation coded as (7).
- *Labor market position* was coded as Working (1; including employed, self-employed or on temporary leave of absence e.g., for parental leave); Studying (2); Age retirement (3); and Non-employment (4; including unemployment; labor market program; long-term sick leave, early retirement; and taking care of household).

Experiences of violence and harassment were measured by:

- *Fear of violence*, which was based on the question ‘Do you ever refrain from walking out alone in fear of being assaulted, robbed or in other way harassed?’ and coded as ‘no’ (0) or ‘yes’ (1) (including ‘yes, sometimes’ and ‘yes, often’).
- *Threat/violence* was based on two items on whether the respondent during the last 12 months had been exposed to physical violence, or threat of violence, so that she/he was frightened, respectively, which were combined into one variable coded as ‘no’ (0) and ‘yes’ (1) (including exposure to violence and/or to threats).
- *Degrading treatment* was based on whether the respondent during the last 12 months had been treated in a way that was perceived as degrading/humiliating, which was coded as ‘no’ (0) or ‘yes’ (1) (including ‘yes, once or twice’ and ‘yes, multiple times’).

Material conditions covered three factors:

- *Cash margin* (whether the respondent would be able to get hold of 15,000SEK (approx. 1,600EUR) in one week), coded as ‘no’ (0) and ‘yes’ (1).
- *Difficulties to make end meet* (whether the respondent had had difficulties paying running costs during the last 12 months), coded as ‘no’ (0), ‘yes, once’ (1) and ‘yes, multiple times’ (2).
- *Residential ownership* (type of residence of respondent), coded as Owned residence (0) (including owned apartment and house); Rental apartment (1) and Other (2) (including live-in, student room, and other living arrangements).

Work conditions comprised two factors:

- *Job dissatisfaction* (‘How well do you enjoy your work tasks?’), coded as satisfied (0), including ‘very good’ and ‘fairly good’; and dissatisfied (1) covering ‘neither good nor bad’; ‘fairly bad’; and ‘very bad’.
- *Job insecurity*, based on the question ‘Are you worried about losing your job within the coming year?’ and coded as ‘no’ (0) and ‘yes’ (1).

Healthcare contacts were based on two questions on whether the respondent considered her/himself to be in need of medical and dental care, but nevertheless refrained from seeking care, during the last three months. A separate item gave the option to provide the reason for the unmet care. These variables were used to construct three variables on unmet *medical* care needs due to

- *Inaccessibility* (including ‘too long waiting times’; ‘difficulties to reach the provider by phone’; ‘too late appointment’; and ‘not knowing where to turn’), coded as ‘no’ (0) and ‘yes’ (1).
- *Negative experiences* (from previous visits), coded as ‘no’ (0) and ‘yes’ (1).
- *Other reasons* (including ‘other reasons’; ‘lack of time’; ‘financial reasons’; and not specified), coded as ‘no’ (0) and ‘yes’ (1).

As well as two variables on unmet *dental* care needs due to

d) *Financial reasons*, coded as ‘no’ (0) and ‘yes’ (1).
e) *Other reasons* (including ‘procrastination or fear of dentist’, ‘not having the time’, ‘other reason’, and not specified), coded as ‘no’ (0) and ‘yes’ (1).

### Data analysis

Complete case analysis was employed in all analyses, with the lowest sample size being N = 22,460 in analyses of general health and all explanatory factors. To illustrate sexual orientation inequalities in health and life conditions, all variables were compared between LGBQ and heterosexual groups, using t-tests (continuous variables) and *χ* ^2^ tests (categorical variables). Auxiliary analyses comparing all four sexual orientation groups (data not shown) showed that each of the three minority sexuality groups displayed worse mental and physical health than the heterosexual group (analysis of variance with Bonferroni post hoc test for multiple pairwise comparisons; *p* < 0.001). They also consistently displayed less favorable profile than the heterosexual group with respect to the majority of the explanatory factors (*χ* ^2^ tests), with exceptions including gender, education, age and job dissatisfaction for which the results were mixed between LGBQ groups.

To address the aims of the study, Blinder-Oaxaca decomposition analysis [24] was used through the *oaxaca* command [25] in Stata 13. Blinder-Oaxaca decomposition analysis, described in detail by O’Donnell et al. [24], aims to attribute a health gap between two groups to the independent contributions of a set of explanatory factors. In the present study, the outcome is mean GHQ-12 and general health score differences between heterosexual and LGBQ sexual orientation groups, which can be expressed as Δ*y* = *y* ^*heterosexual*^ *– y* ^*LGBQ*^ *.* The method, is based on two linear regression models that are fit for each of the groups, and which in the case of the present study can be expressed as:

$$ {y_i}^{heterosexual}={\beta}^{heterosexual}{x}_i+{\varepsilon}^{heterosexual} $$

and

$$ {y_i}^{LGBQ}={\beta}^{LGBQ}{x}_i+{\varepsilon}^{LGBQ} $$

where the vectors of the *β* parameters include intercepts. The health gap Δ*y* is then expressed as derived from differences in the explanatory factors *x*’s (Δ*x*), and from differences in regression coefficients (Δ*β*), which in the single predictor case can be described as follows:

$$ \varDelta y=\varDelta x{\beta}^{LGBQ}+\varDelta \beta {x}^{LGBQ}+\varDelta x\varDelta \beta $$

where the first terms represents the group difference in the explanatory factor *x (*Δ*x,* referred to as *the explained part*) weighted by the coefficient of the LGBQ group (*β* ^*LGBQ*^ *)*; the second term represents the group difference in the coefficient (Δ*β,* labelled *the unexplained part*) weighted by the *x* of the LGBQ group (*x* ^*LGBQ*^), and the third the interaction between the difference in *x* (Δ*x*) and the difference in *β* (Δ*β*) [26]. For a predictor to be able to make a substantial independent contribution to the sample-level health gap, it needs to be related to the health outcome, be unequally distributed by the social indicator, and be of a sufficient sample frequency.

In the present study, the health gaps between heterosexual and LGBQ groups were decomposed separately for GHQ-12 and general health complaints, with all explanatory factors described above added to explain the health gaps. Model estimates indicate how well the explanatory factors together explain the total health gap, and are reported as total explained (the sum of contributions of all explanatory factors) and unexplained fractions, which are expressed in both absolute (on the same scale as the outcome), and relative (percentage of the absolute total health gap) terms, with p values. Contributions of each individual explanatory factor to the observed health gap are similarly reported as absolute and relative contributions with *p* values, but for which relative contributions are relative to the absolute *explained* fraction rather than to the total health gap. The *normalize* subcommand was use to summarize the total contribution of all categories of each categorical variable, which are reported in the results section.

## Results

### Life conditions of LGBQ and heterosexual people

Table 1 displays the characteristics of the sample by sexual orientation. In total, 844 persons (3.5%) reported LGBQ and 23,446 (96.5%) heterosexual orientation. People with a LGBQ orientation reported worse mental health and general health complaints compared to the heterosexual group, thus demonstrating sexual orientation-based inequalities in self-assessed health. Lesbian-Gay-Bisexual-Questioning people were overall younger rather than middle-age, although the fraction in older age was similar between the groups, as was the gender distribution. They had slightly lower education, less qualified jobs and considerably lower income, were more commonly studying or non-employed than working, reported worse material conditions, and higher job insecurity despite similar satisfaction at work. They reported more fear of violence in public space, and a more than double frequency of exposure to threat or violence. Degrading treatment was reported in more than one third of those of sexual minority, compared to less than a sixth among those of heterosexual orientation. Furthermore, the LGBQ group also reported unmet medical and dental health care needs about twice as often as the heterosexuals.

**Table 1:** Descriptive statistics of all included variables by sexual orientation

Variable	LGBQ		Heterosexual		Difference
	*N*	%	*N*	%	*P* value
Mental Health, M (SD)	23.48	6.39	21.29	4.58	<0.001
Health complaints, M (SD)	14.95	3.66	13.95	3.03	<0.001
Women	480	56.9%	12546	53.5%	0.052
Born outside Sweden	94	11.1%	1451	6.2%	<0.001
Age					<0.001
- Young adult	375	44.4%	5403	23,0%	
- Middle-age	221	26,2%	10797	46.0%	
- Older	248	29.4%	7258	30.9%	
Income					<0.001
-Quintile 1	342	40.9%	4444	19.0%	
-Quintile 2	210	25.1%	4379	18.7%	
-Quintile 3	133	15.9%	4754	20.3%	
-Quintile 4	78	9.3%	4872	20.8%	
-Quintile 5	74	8.8%	4939	21.1%	
Education					<0.001
-Low	453	57.9%	11018	48.1%	
-Medium	244	31.2%	7888	34.4%	
-High	85	10.9%	4005	17.5%	
Occupational class					<0.001
-Service class	47	5.6%	2069	8.8%	
-Routine non-manual	90	10.7%	4639	19.8%	
-Petty Bourgeoisie	45	5.3%	2087	8.9%	
-Assistant non-manual	60	7.1%	2664	11.4%	
-Skilled manual	127	15.0%	4490	19.1%	
-Unskilled manual	237	28.1%	4959	21.1%	
-Unspecified	238	28.2%	2550	10.9%	
Labor market position					<0.001
- Working	273	32.9%	12308	53.1%	
- Studying	182	21.9%	1691	7.3%	
- Retired	236	28.4%	6863	29.6%	
- Non-employed	139	16.7%	2317	10.0%	
Fear of violence	168	20.3%	2944	12.8%	<0.001
Threat/violence experience	87	10.6%	915	4.0%	<0.001
Degrading treatment	286	34.8%	3623	15.8%	<0.001
Diff. make ends meet					
-Sometimes	71	8.5%	1151	4.9%	<0.001
-Often	118	14.1%	1316	5.6%	<0.001
Low cash margin	323	38.7%	3572	15.3%	<0.001
Residential ownership					<0.001
-Resident-owned	446	53.4%	17675	75.7%	
-Rental	247	29.6%	4116	17.6%	
-Other arrangements	142	17.0%	1560	6.7%	
Job dissatisfaction	74	8.8%	1725	7.4%	0.121
Job insecurity	103	12.2%	1813	7.7%	<0.001
Unmet medical needs: inaccessibility	98	11.6%	1099	4.7%	<0.001
Unmet medical needs: experiences	87	10.3%	1288	5.5%	<0.001
Unmet medical needs: other	128	15.2%	1903	8.1%	<0.001
Unmet dental needs: other	131	15.5%	1965	8.4%	<0.001
Unmet dental needs: economical	117	13.9%	1662	7.1%	<0.001

*LGBQ* lesbian-gay-bisexual-questioning orientation

Overall, the descriptive results thus pointed to worse health in combination with a ubiquitous material and psychosocial disadvantage among those of LGBQ orientation, compared to those of heterosexual orientation.

### Decomposition of sexual orientation inequalities in health

**Fig. 1:**
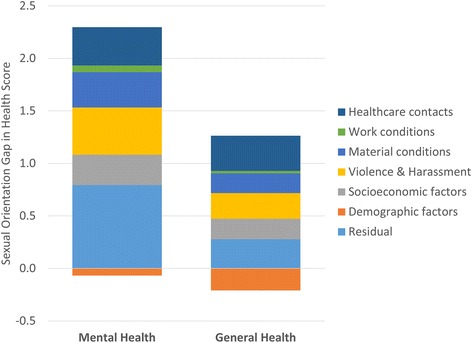
Contributions of factors to health inequalities between lesbian/bisexual/gay/questioning vs heterosexual orientation groups

In the next step, in an attempt to attribute the observed sexual orientation-based inequalities in mental health and general health complaints to independent contributions of the inequalities in life conditions described above, the life conditions were used as explanatory factors in Blinder-Oaxaca decomposition analyses for mental and general health. See Table 2 for a summary of the results, showing the total fraction explained of the health gap, as well as the contribution of each explanatory factor; and Fig. 1 for an illustration of the joint contribution of groups of explanatory factors to the absolute health gaps.

**Table 2:** Summary of Blinder-Oaxaca decomposition analyses of health gaps between sexual orientation groups

Model estimates	Mental Health			Health Complaints		
	Abs.	Rel. (%)	*P*	Abs.	Rel. (%)	*P*
Mean health (LGBQ)	23.491		<0.001	15.003		<0.001
Mean health (heterosexual)	21.263		<0.001	13.949		<0.001
Mean health difference	2.228		<0.001	1.054		<0.001
Explained fraction	1.435	64.4	<0.001	0.776	73.6	<0.001
Unexplained fraction	0.793	35.6	<0.001	0.278	26.4	0.016
Contributions						
Gender	0.017	1.2	0.022	0.032	4.2	0.014
Country of Birth	−0.011	−0.8	0.057	0.003	0.3	0.451
Age	−0.075	−5.2	<0.001	−0.244	−31.4	<0.001
Income	0.036	2.5	0.101	0.079	10.2	<0.001
Education	−0.013	−0.9	0.106	0.039	5.0	<0.001
Social class	0.056	3.9	0.010	0.014	1.8	0.289
Labour market position	0.211	14.7	<0.001	0.063	8.1	0.010
Fear of violence	0.050	3.5	<0.001	0.048	6.2	<0.001
Threat/violence experience	0.035	2.4	0.005	0.011	1.5	0.069
Degrading treatment	0.366	25.5	<0.001	0.186	24.0	<0.001
Diff. make ends meet	0.172	12.0	<0.001	0.086	11.1	<0.001
Low cash margin	0.135	9.4	<0.001	0.110	14.2	<0.001
Residential ownership	0.029	2.0	0.158	−0.010	−1.3	0.394
Job dissatisfaction	0.022	1.5	0.400	0.008	1.1	0.374
Job insecurity	0.043	3.0	0.001	0.015	1.9	0.004
Unmet medical needs: inaccessibility	0.096	6.7	<0.001	0.075	9.7	<0.001
Unmet medical needs: experiences	0.073	5.1	<0.001	0.099	12.8	<0.001
Unmet medical needs: other	0.120	8.4	<0.001	0.098	12.6	<0.001
Unmet dental needs: other	0.030	2.1	0.004	0.027	3.5	<0.001
Unmet dental needs: economical	0.046	3.2	0.001	0.035	4.5	<0.001

*LGBQ* lesbian-gay-bisexual-questioning orientation

Estimates shown are absolute (Abs.) and relative (Rel.) contributions and *p* values

As can be seen in Table 2, the explanatory factors jointly explained a significant and considerable portion of the observed health gap; 64% in mental health and 74% in general physical health. The single most important factor for both health inequalities was degrading treatment, which explained 24-26% of the explained part of the health gaps. Fear of violence made a smaller independent contribution, and despite the substantial difference in exposure to threats/violence (Table 1), this factor had less importance in explaining the health gaps, likely a reflection of it being fairly uncommon in both sexual orientation groups.

In addition to the experiences related to violence and harassment, certain socioeconomic and material factors - including difficulties to make ends meet, low cash margin, labor market position and for health complaints also income - jointly explained as much as 38-45% of the health gaps. The category-level contribution of labor market position (not displayed in table 2) was largely attributable to non-employment, alone explaining 8.5% of mental and 12.1% of general health, with studying contributing to the sexual orientation gap in mental (6.8%) but not general (−3.1%) health. For difficulties to make ends meet, the greatest contribution came from the most disadvantaged category, explaining 9.7-9.8% of the health gaps. Another substantial contribution came from health care contacts, which jointly explained 43% in the health complaints and 25% in the mental health gaps; mostly attributed to unmet medical care needs but with smaller significant contributions also from unmet dental care needs.

None of the demographic factors contributed majorly to the observed health inequalities. In contrast, age displayed an offsetting contribution to the inequality (as seen by the negative contribution) particularly for health complaints, as a reflection of young adults more frequently reported LGBQ orientation (Table 1) but simultaneously reported better health, compared to those in middle-age.

## Discussion

The present Swedish study is one of the few population-based studies aiming to comprehensively explain health inequalities between sexual orientation groups. In accordance with minority stress theory, the study shows that the health inequalities between LGBQ and heterosexual people can, to a substantial degree, be attributed to the differential risks of psychosocial exposures such as degrading treatment in everyday life. The results also suggest that several distinct areas of life – including labor market position, access to financial resources and navigating the health system – each are as important as degrading treatment to independently explain health inequalities between LGBQ and heterosexual people. This finding rather support fundamental cause theory, and emphasizes that structural-level discrimination limiting LGBQ people’s access to material resources in multiple societal arenas may be key to understand the underpinnings of sexual orientation based inequalities in Sweden.

Although previous studies on sexual orientation based inequalities in health traditionally have focused on inequalities in mental health [1, 4, 27–30], reports on physical or general health inequalities in Sweden and other settings [2, 7, 15, 31, 32] have increased in recent years. Our study confirms inequalities in both self-assessed mental and physical health inequalities by sexual orientation, and adds that multiple psychosocial and material factors appear to be of importance for both inequalities, with unmet care needs and socioeconomic conditions possibly playing a greater independent role for physical health inequalities.

Previous Swedish reports [2, 15] highlight overt discriminatory experiences, such as violence and perceived discrimination, as important for sexual orientation inequalities in health. The present study did not differentiate between different sources of discrimination, e.g., due to sexual orientation or gender or ethnicity, but supports the importance of degrading treatment more broadly for sexual orientation inequalities in health, by using the suitable statistical approach of decomposition analysis. Our findings thereby corroborate what is set out by minority stress theory [4]; that those of minority sexual orientation display worse health at least partly due to differential exposure to psychosocial stressors, such as degrading or humiliating treatment.

However, the most important contribution of the present study is that unequal distribution of material resources – as indicated by financial privilege, labor market position and health care access - were as important as the more established psychosocial stressors in explaining the sexual orientation inequalities in health. Underutilization of health care among LGBQ persons, including refraining from healthcare for financial reasons, have been reported before in the US [17], and shows how intertwined different material indicators may be. This facet of the present study suggests that the sexual orientation inequalities in health are not merely explained by stressor exposure, but underpinned by ubiquitously dispersed social inequalities between sexual orientation groups, spanning across multiple spheres of life. This pattern is less supportive of minority stress theory and more supports the tenets of fundamental cause theory [6, 7], which posits that the unequal distribution of a range of flexible protective resources, such as money, social capital, and power, can be utilized in order to gain a health advantage.

From an ecosocial standpoint [9, 10] the findings can thus be understood as social inequalities which, by pathways spanning multiple levels and spheres of social organization, end up embodied in the form of poor health and thus produce and reproduce the population-level health inequalities between LGBQ and heterosexual people. Our study thereby paints a worrisome picture of pervasive discrimination operating in the lives and becoming expressed in the body of Swedish LGBQ people, above and beyond what can be readily recognized as a discriminatory experience for the individual. As put by Krieger ‘bodies tell stories that people cannot or will not tell, either because they are unable, forbidden, or choose not to tell’ [33]. This calls for a more comprehensive approach to the foundations of sexual orientation inequalities in health.

When it comes to the human rights situation for LGBQ people in Sweden and the country’s declining position in the ILGA’s European rankings in recent years [11, 14], it is important to note that such international rankings seldom take into account material preconditions, such as access and funding of healthcare, unemployment benefits and social welfare; welfare systems which also more generally have become eroded in Sweden in recent years [34]. Access to welfare systems can be of particular importance for LGBQ people, who may face for example discrimination on the labor market, be at higher risk of mental illness and who may need medical assistance to become parents, aspects which this study suggests are also salient for health inequalities. Mirroring our suggestion for research on health inequalities, our findings therefore additionally implies that the material living conditions of LGBQ people ought to be considered in international assessments and social and public health policy, lest important facets of the lives of LGBQ people which are most materially expressed in poorer health risk remaining unnoticed and unconsidered.

### Methodological considerations

The strengths of the study include a large population-based study population, multiple sources of linked data, and the use a novel statistical methods suitable to the question at hand.

The study was cross-sectional and as such causal inferences are hazardous. The response rate was only about 50%, which may severely bias point estimates of frequencies, such as the proportion of LGBQ/heterosexual sexual orientation. Lacking regionally comparative survey data on attitudes towards LGBQ people, it is possible that Northern Sweden differs compared to the rest of Sweden, which would limit generalizability. Nevertheless, the frequencies of LGB (1.8%) and LGBQ (3.5%) found in the present study match the frequencies from other population studies from Sweden (2.0-2.3% LGB and 3.2% LGBQ [2, 7, 15]), as well as from the US (1.6-2.4% LGB [17, 35]) and the Netherlands (2.8% LGBQ [32]). Moreover, it is less likely that the point estimates of health differences between these groups, or the explanatory value of individual factors, are as severely affected by potential selection bias.

Sexual orientation was defined binary as either heterosexual or LGBQ, in line with the point of departure of minority stress theory, as a necessity imposed by the analytic approach requiring binary exposure, and due to the low frequencies of specific minority status groups. Nevertheless, we acknowledge this may be a conceptually and empirically crude approach [28], which also may conceal important heterogeneity in health inequalities, e.g., by specific sexual orientation groups [15], age and gender [2, 7], or by education [35]. For example, complementary analyses showed that the three sexual minority groups did not differ in a uniform direction from the heterosexual group with respect to certain variables, which means that these factors could be of greater importance for specific sexual minority groups. However, the fact that the three sexual minority groups consistently reported worse health and for the majority of indicators also worse life conditions than the heterosexual group, gives validity for the crude binary formulation.

In addition to the crude formulation of sexual orientation groups, several of the explanatory variables have similar limitations. For example, country of birth was due to low frequencies operationalized as a simple binary variable, and age analyzed in large age groups according to life course period. Sensitivity analyses with a three-level country of birth variable and with continuous age did however not change the inferences. Furthermore, the measure of degrading treatment was a simple binary variable and did not differentiate between possible reasons for the treatment, e.g., discrimination due to sexual orientation or ethnicity or gender, but as argued in other parts of the manuscript, it may be impossible for an individual to accurately discern the structural roots of a humiliating experience. Here it is also important to note we controlled for several indicators which could be alternative sources of discrimination, such as country of birth, gender, socioeconomic factors and employment.

Concerning the analysis, the sexual orientation groups were of very unbalanced sample size. Group-level matching procedures would have been possible to render the groups more similar in size, but would run the risk of concealing and underestimating the health inequalities and their underpinnings. The approach of the present report was therefore to instead attribute the inequalities to a comprehensive set of factors, including to demographic factors which would rather be considered confounders, such as age, country of birth and gender. Decomposition analyses are illustrative to partition the independent contributions to a given health inequality, but do not illustrate processes or support causal inference to any greater degree than conventional regression models. To mitigate entanglement with the health outcomes, we intentionally focused on ‘environmental’ exposures rather than proximal mediators such as behaviors or emotions which might be considered part of, or consequences of, particularly poor mental health. Nevertheless, processes of mediation are likely among the factors studied, which might lead to lower estimates for causally distal factors, such as socioeconomic conditions. Some factors could also represent consequences rather than causes of health. As a pertinent example, unmet health care needs can plausibly lead to poorer health, but since it also requires a health care need to begin with, it is also influenced by initial health status. It is therefore important to bear in mind that the results only refers to the *independent* contributions of each factor considered, using a cross-sectional design, and with any statement concerning causality ultimately a tentative hypothesis.

## Conclusions

The present study demonstrates that mental and general physical health inequalities between heterosexual and LGBQ people in the reputably fairly ‘gay-friendly’ setting of Sweden may only partially be explained by overt and identifiable experiences of harassment and discrimination, but where other structural barriers such as financial disadvantage, discrimination on the labor market and poorer access to health care each may be just as important. These findings suggest that pervasive discrimination underpins much of the sexual orientation based inequalities in health in Sweden. A greater consideration of such structural-level discrimination should be heeded in international assessment and monitoring the living situation for LGBQ pepple, and in research and social and public health policy working towards equity in health between LGBQ and heterosexual people.

## Abbreviations

EUR: Euro
GHQ-12: General Health Questionnaire-12
HET: Health on Equal Terms
ILGA: The International Lesbian, Gay, Bisexual, Trans and Intersex Association
LGBQ: Lesbian/Gay/Bisexual/Questioning
SEK: Swedish Krona
